# Discourses on the postcoital pill in young women 

**DOI:** 10.1186/s12889-018-5691-3

**Published:** 2018-06-27

**Authors:** M. L. Bauzà-Amengual, M. Esteva, M. Ingla-Pol, M. A. Font-Oliver, S. March

**Affiliations:** 1 Department of Nursing and Physiotherapy, Universitat Illes Balears, Carretera de Valldemossa, km 7,5, 07122 Palma, Illes Balears Spain; 2 Research Group, Cancer Preventative Action, Carretera de Valldemossa, km 7,5, 07122 Palma, Illes Balears Spain; 3 University Research Institute in Health Sciences, Carretera de Valldemossa, km 7,5, 07122 Palma, Illes Balears Spain; 4 Balearic Islands Health Research Institute (iDisBA), Hospital Universitari Son Espases, Edifici S. Carretera de Valldemossa 79, 07120 Palma, Illes Balears Spain; 5 Unit for Research, Primary Health Care Management, Mallorca, IB-Salut, Carrer de la Reina Esclaramunda, 9, 07003 Palma, Illes Balears Spain; 6 Health Promotion Agency, Palma Town Hall, Plaça de Cort, 1, 07001 Palma, Illes Balears Spain

**Keywords:** Contraception, Postcoital, Qualitative research, Interviews as a theme, Sex education

## Abstract

**Background:**

Emergency contraception (EC) is an effective postcoital contraceptive method for reducing the risk of unwanted pregnancy after unprotected intercourse. The estimated effectiveness of EC is between 70 and 89% if taken within 72 h following intercourse. Most of the studies carried out in Spain are quantitative and from the perspective of health professionals. In this study, we intend to explore the knowledge of, attitudes towards and discourse regarding the use of EC in women aged 15 to 25 years.

**Methods:**

Sample: A qualitative study including in-depth interviews with 19 women between 15 and 25 years of age was performed. Inclusion criteria: Participants were natives of Spain or of a Latin American country. Segmentation criteria: Participants had experience in the use of EC.

**Data collection:**

Participants were selected by health care informants and by the snowball technique among university students.

Data analysis: A thematic analysis was performed. Preliminary analyses were made during the course of the field work to adapt the script and to assess data saturation. A preliminary code tree was developed by two researchers, and the coding of the text was done with the Atlas.ti 5.0 software.

**Results:**

EC is perceived positively by women. They do not express issues with taking it, although some feel guilty. The reason for taking EC is to avoid unwanted pregnancy and abortion. Women also feel that EC should be used in moderation. False beliefs and misconceptions regarding EC are held: EC delivers an excess of hormones, induces abortion and causes severe side effects. Women mention that the health professionals who provide EC have moral beliefs. Women use it because of condom breakage associated with their first coital relations.

**Conclusions:**

The results of this study have public health implications: The sexual-affective health education received by young people should incorporate clear information about the mechanism of action of the EC pill and its side effects together with empowerment strategies addressing guilt and moralistic messages. Programmes and training activities for health professionals must be designed to prevent the communication of inappropriate messages such as those that exaggerate the side effects of EC and those that promote fear and guilt, because they represent a barrier to the responsible use of this medication.

## Background

Emergency contraception (EC) is an effective postcoital contraceptive method geared towards reducing the risk of unwanted pregnancy after unprotected intercourse or contraceptive failure. It has been estimated that its effectiveness is between 70 and 89% if taken within 72 h after intercourse [1]; its effectiveness decreases after that period. Over the past few decades, in an attempt to reduce unwanted pregnancies, governments in developed countries have made efforts to expand women’s access to emergency contraceptive methods using mechanisms such as its dispensation as an over-the-counter medication [2] or through the anticipated delivery of the drug [3–7]. In Spain, over-the-counter dispensation in pharmacies began in 2009 [8]. These methods of increasing women’s access to EC are of positive value because they decrease the amount of time necessary to obtain it, thereby ensuring its effectiveness [9]. On the other hand, over-the-counter availability of EC helps to overcome inconveniences such as the embarrassment of having to consult a health professional, thus encouraging its use in women who are reluctant to seek consultation; EC can be obtained with greater anonymity when it is available directly through the pharmacy [6]. In countries where access to the EC pill has been deregulated for many years, abortion rates have not declined as was anticipated by the drug legislation policies. Glasier [3] performed a community study whose results showed that the widespread distribution of EC pills through health services did not decrease the abortion rate in the UK. Numerous reasons may have contributed to the lack of effect of EC availability on the decline in unwanted pregnancies. Among these reasons is the persistence of erroneous beliefs caused by the lack of knowledge about the mechanism of action of EC [10]. The belief that EC is an abortion-inducing drug as well as the exaggeration of the side effects causes some women to decide not to take it [10, 11]. In addition, factors such as the cost and the sense of intimidation generated by the possible moral judgements of the health professionals or pharmacists who dispense the pill must be considered [10, 12, 13]. In other cases, women state moral or religious reasons for not using EC, while others decline use because they think or expect that they will not get pregnant [12].

The majority of studies on this subject performed in Spain to date applied quantitative approaches, and most of those were carried out from the point of view of health practitioners. It is therefore necessary to explore the conceptions regarding EC that are elicited from the discourse of women who are users or potential users. By incorporating the perspective of young women, we aspire to improve the success chance of public policies aimed at promoting sexual-affective health. A necessary first step is the identification of the cognitive barriers that will allow the conceptual errors underlying the formation of unfavourable attitudes towards EC to be debunked. This knowledge may also be useful in understanding the discourses that lead some women to use EC. In this study, we intend to explore the knowledge of, attitudes towards and discourses regarding the use of EC in women aged 15 to 25 years.

## Methods

This investigation is a qualitative study applying in-depth interviews. The study aims to unveil the discourses regarding the contraceptive pill among young women living in Mallorca, Spain.

### Population and theoretical sampling

The study population was composed of young women between the ages of 15 and 25 of different socioeconomic levels who fulfilled the following inclusion criteria: a resident of Mallorca, and a native of Spain or of a Latin American country.

A purposive sampling technique was used to ensure that women both with and without experience in EC use were included in the study. In addition, the following criteria were used to maximize sample diversity: level of education, occupation, age group and partnership status. Young women in Latin America have a high rate of voluntary abortion compared to native Spaniards. Therefore, to compare the discourses, both Spanish and Latin American women were interviewed.

A total of 19 interviews were conducted until preliminary analysis identified the saturation of data regarding the study objectives. Even women who had no personal experience in the use of EC were aware of other experiences through their friends and were thus included in the study group.

### Data collection

Information was collected through in-depth, individual, semi-structured interviews. The choice of technique was based on the intimate nature of the interview topic and the ability of this method to facilitate adaptation to the schedules and locations of the persons interviewed. For the preparation of the interview script (see Table 1), the literature was reviewed and contrasted with responses from key informants. The key informants were primary health care nurses who assisted young women in health promotion programmes developed in high school centres or in gynaecological consultations. The scripts were adapted during the course of the fieldwork using the results of the preliminary analysis. The participants were intentionally selected by the key informants in the health centres and by a university nurse lecturer, based on the identification of women who met the inclusion criteria and were likely to participate. The lecturer did not recruit her own students but instead asked them to invite other students to participate. Before the interviews were conducted, the key informants were contacted by the research team to facilitate the fieldwork. The key informants collaborated in recruiting the women to be interviewed during face-to-face encounters in their work locations. Once participants were identified, the objectives of the study were explained to them and they were asked to sign an informed consent document. After the consent document was signed, the research team scheduled the interviews. No selected individual refused to participate.

**Table 1:** Script of the semi-structured interview

1. - Practice of sexual relations and use of contraception.	
• With whom do you talk about sexuality?	
• What is the meaning of sexual relations to you?	
• How you have been educated?	
• Have you had sex with penetration?	
• What do you think about contraceptives, and what is your personal experience with contraceptives?	
2. - Pregnancy and abortion.	
• Opinion about when and under what circumstances you should be a mother.	
• Opinion on abortion and personal experience with abortion.	
3. - Emergency contraception.	
• What do you know about the morning after pill?	
• Information about your own experience and your environment.	
• Opinion on and experience with the dispensation of EC.	
• Exploration of false beliefs.	

Three female researchers who had previously been trained in the interview technique and script and who did not previously know the interviewees conducted the interviews. The emphasis throughout the interviews was empathic rather than moralistic. Interviews were performed in environments chosen by the interviewees to promote intimacy. All interviews were tape-recorded and were subsequently professionally transcribed verbatim.

### Data analysis

We performed a thematic analysis of the discourse collected in the transcripts. The model of analysis used is a hybrid approach of inductive and deductive coding [14] with a previous coding tree, but open to additions of emerging categories or themes. Raw data was coded using the Atlas.ti 5.0 software in accordance with an ex ante code tree developed by two researchers on the team. During the coding process, emergent categories were progressively evident and the code tree was adapted accordingly. This way, we finally use 36 codes to label the raw data. From the process of connecting the codes and looking for patterns in the data, emerged 5 main themes which portrayed the phenomenon studied: The conception of sex and sexuality, the ideas about abortion, the perception and knowledge about EC, the reasons for using it and the barriers to using it. These topics are grouped into three large blocks in the results.We performed preliminary analyses during the course of the fieldwork to adapt the script and to assess data saturation. Differences in discourses between EC users and non-users were explored. The results were discussed and interpreted by the entire research team and were presented to selected participants in order to validate the data by triangulation.

## Results

### Population data

The study population consisted of 19 women between the ages of 15 and 25. The descriptive characteristics of these women are shown in Table 2. Women of Latin American origin comprised 21% of the participants, while the remainder were natives of Spain. A large majority were students: one was a student enrolled in compulsory education, while the others were high school or university students. Most participants reported having a stable partner and only one of them was a mother. Five women reported having taken EC.

**Table 2:** Descriptive characteristics of the sample

INTERVIEW N°	Age	Country of Birth	Occupation	Level of Education	Sexual Experience	Partner	Children	Use of EC
E1	20	Spain	Unemployed	Junior High School	No	Yes	No	Yes
E2	25	Spain	Physical Therapist	University	No	Yes	No	No
E3	25	Cuba	Unemployed	High School	Yes	Yes	2	Yes
E4	18	Spain	Unemployed	High School	No	Yes	No	No
E5	19	Colombia	Student	University	No	Yes	No	No
E6	25	Spain	Mobile Phones	Professional Training	No	Yes	No	No
E7	20	Spain	Unemployed	High School	Yes	No	No	No
E8	16	Ecuador	Student	High School	No	No	No	No
E9	25	Spain	Environmental Consultant	University	No	No	No	Yes
E10	24	Spain	Clerical/ Student	University	Yes	Yes	No	Yes
E11	24	Spain	Nursing Assistant	Professional Training	No	Yes	No	No
E12	21	Spain	Student	University	No	Yes	No	No
E13	22	Spain	Student	University	No	No	No	No
E14	17	Ecuador	Unemployed	High School	No	Yes	No	No
E15	20	Spain	Student	University	No	No	No	Yes
E16	15	Spain	Student	High School	No	Yes	No	No
E17	17	Spain	Student	High School	No	No	No	No
E18	15	Spain	Student	High School	No	Yes	No	No
E19	20	Spain	Unemployed	High School	Yes	Yes	No	No

### Conception of sexuality and abortion and data regarding sexuality

In exploring the meaning that sexual relations have to the interviewed women, we found that the discourses showed that sexual relations are associated with confidence in a partner, with love, and with friendship; and are seen as a way of showing affection. These discourses appeared more frequently among younger women.

*Question (Q): “For you, what does it mean to have sex?”*

*Answer (A): Feelings, love.*

*Q: Yes.*

*A: And that’s it.*

*Q: And that’s it.*

*A: (laughs).*

*Q: And need? No.*

*A: No.* (E3, 25 years old).

However, some women, in addition to the discourses described previously, also introduced the concept of sex linked to pleasure and separated from love.

*A: “I have a conception that I do not care about love and sex, because I consider them separate. One thing is for pleasure and the other... Obviously you always have to have confidence. But love and pleasure are not always linked... That is, with your partner you can also share this, which is one of the most important parts, but the person you have sex with does not have to be your partner. And it is even more frequent. There are more and more cases, and I think it’s the right thing to do because you do not have to have to tie yourself to a person with whom you only feel desire”.* (E15, 20 years old).

In exploring the discourses about the participants’ views on abortion, we found widespread consensus in favour of abortion. Despite the strong social and emotional burden that condemns the act, the surveyed women would generally abort an unwanted pregnancy, although they classified it as a final option. It was observed that women try to overcome the contradiction that the abortion poses to them by defining the conditions in which this practice seems more acceptable. These conditions included a malformation or serious illness of the foetus, or the possession of inadequate financial resources to educate the child.

*A: “Because a religion, say, is something that invites you not to abort because they tell you, because it is your child, because it is the fruit of I do not know what and well, okay. I believe, I am a believer and I know perfectly the values that you are supposed to have, but you will not be able to have a child being a teenager. First, it’s going to be very expensive for your parents and second, you will not know how to take care of it and, I do not know, I do not see it. I would prefer to abort because I know that that son will not be in the best conditions and to have it that way, no.”* (E5, 19 years old).

Regarding the information obtained about sexuality issues, most women reported that, to this day, these issues remain taboo within families. The peer group (friends) continues to have the greatest influence in socializing these topics. In addition, some women reported that the information they have received about these subjects has been provided in school.

*Q: “At home, have your parents talked to you about contraceptives and things about sex?”*

*A: No.*

*Q: And the information you have, from where did you get it? From friends, school...?*

*A: Yes, from friends and school.*

*Q: Yes? They have spoken to you of the subject in school?*

*A: Yes, one day, yes.* (E18, 15 years old).

Note that for those women who were sufficiently confident to talk about sexual aspects at home, the mothers rather than the fathers are the adult reference for these issues.

A: “*Not with my father. And with my mother, yes. My mother remembered that I started talking about sex when I first got my period and she spoke to me from a very scientific point of view, let’s say, you have to use a condom and all that, but we have never spoken openly for example of masturbation or sexual pleasure or things like that, instead we’ve talked things like... you have to use a condom...”* (E10, 24 years old).

### Perceptions and knowledge related to EC

All women interviewed reported hearing about the postcoital pill, even women who had not used it. They agreed that it is preferable to have access to EC rather than to have an abortion. They concluded that they have considered the option of EC and either have taken it or would take it out of a fear of pregnancy resulting from risky coitus. The issue that worried them the most was an unwanted pregnancy. In contrast, the discourse about protection against sexually transmitted diseases was not reported. Although all interviewees affirmed that they would not be reluctant to use EC, some commented that they would feel bad or even guilty about using it.

*“No, but what is happening is that there is this thing that is a bit weird, you know? And, I do not know, I may have taken it... six times or something like that and a friend of mine told me that she could only take it once every year, you know? Like there’s something scary about it.”* (E10, 24 years old).

In exploring the participants’ knowledge about how to take EC, we found that most women know that such medication should be taken within 72 h of unprotected intercourse and that its effectiveness decreases the longer the woman waits to take it.

*“... I think it’s three days or five, I do not know very well, after having sex, but it’s best to take it as soon as you’ve had sex and you’ve had some risk or something. You will not let go a month by knowing that you may have had something, and to have problems later.”* (E5, 19 years old).

Regarding the side effects that EC can produce, some women mentioned that they have heard that EC can cause dizziness and/or vomiting and that the overuse of EC should be avoided because it contains an excess of hormones.

*“...Is like a thing that’s a little extreme because it’s a hormonal bomb for your body, I do not think any woman wants to take it.”*

*Q: Do you think the pill has side effects?*

*A: Yes.*

*Q: Like?*

*A: “I think a hormonal imbalance.”* (E10, 24 years old).

We also found references to the belief that EC is associated with abortion, although the nature of this relationship was not completely clear from the participants’ discourses and may contradict reality.

*Q: “Do you think it’s an abortion pill?”*

*A: “Yes, because it does...that is, if you are pregnant...if you are not sure of being pregnant, you better take it. And yes, if you are pregnant, you kill the child.”* (E16, 15 years old).

We found that most of the interviewed women doubt the abortive nature of EC, but the desire to avoid an unplanned pregnancy overrides the concern about whether EC causes abortion.

### Reasons and barriers regarding EC

In exploring the main reasons behind the participants’ use of EC, we found that the main reasons are condom breakage or slippage. These accidents were most commonly related to the participants’ first sexual experiences. Some interviewees said that either they or their friends have used EC because they had intercourse without using any contraceptive method. The participants also identified this behaviour with their first coital relations.

*“Well, two times I used it because I had two broken condoms. When I started having sex, I still did not know or did not control how you put it on and such. Then, one time, because I did not put it on and another because it stayed inside.”* (E1, 20 years old).

Furthermore, we discovered that some participants used EC for prevention of pregnancy because they were unsure whether they had had protected coitus. In most cases, this uncertainty was linked to alcohol or cannabis use.

*“I used it for safety because we had doubts whether a condom had broken or not, and I took it for safety*.” (E3, 25 years old).

*“For that very reason, they were high on weed or had been drinking and it did not occur to them to use any contraceptives and, just in case, they went on to take it”.* (E6, 25 years old).

With one exception, all of the women interviewed who had taken EC the day after intercourse had taken it several times. Almost all of the interviewees had said that partners participate in the event, and even affirmed that the cost had been shared with their partner. The standard discourse was that the women make the decision of whether to use EC, and the women also involve their partners by requesting that the cost of EC be shared.

*A: “Yes, evidently, evidently, and he paid half each time, no way!”* (E15, 20 years old).

When asked to identify the location from which EC was obtained, the participants stated either the health centre or the pharmacy. Furthermore, two of the interviewees indicated that health professionals in both the pharmacy and the health centre expressed moralistic messages about the use of EC and even expressed misleading messages about the number of times that EC can be taken.

*A: “Yes, I was told that in theory you could not take more than once in a lifetime”.* (E3, 25 years old).

*A: “They gave me a pro-life speech and that it had to be what God wanted.”* (E9, 25 years old).

No differences *of discourses between* EC users and non users have been detected. This is explained because all the women interviewed who did not have experiences of using EC, had experiences of close friends who had used them.

## Discussion

Our study shows that the women interviewed perceive EC as positive and that neither they nor their friends have issues with taking it, although some women admit to experiencing feelings of guilt. The main reason given for using EC is to prevent pregnancy. At the same time, false beliefs and misconceptions regarding EC are evident, especially those regarding its abortive effect and the exaggeration of its adverse effects. Participants stated that EC had been used because of condom-related accidents and less frequently because no contraceptive method was used. In general, the drug had been obtained either in health centres or pharmacies; some women reported that moralistic comments were made by the professionals who dispensed EC.

We have compiled information regarding the conceptions of the women in our study about sexuality and sex education for use as a point of reference when interpreting the discourses. We note that younger women have a discourse relating sex to love and that they associate sex with stable partners. However, the older women and college graduates in our study tend to believe that sex is associated primarily with pleasure and that it is dissociated from love. The concept of romantic love, as much because of its internal conceptualization as because of the external experience of learning about loving others that it promotes, is considered by diverse authors to be an intrinsic part of the social subordination of women [15]. Thus, this study shows that this conception of romantic love continues to be reproduced in the current generation of young women.

The peer group (friends) remain the primary source of socialization related to sexuality. Thus, Esteban (2008) affirms that networks of friends are often privileged spaces of sociability, reciprocity and encouragement for change [16]. The role of mothers as transmitters of knowledge and attitudes about sexual-affective education is important to highlight, although our results seem to indicate that such education remains a pending subject in our society.

All of the women interviewed are aware of EC even if they have not used it and understand that the pill must be used within 72 h of intercourse to be most effective. These results coincide with those of similar studies [17, 18]. Fontes [19] concludes that because EC is used correctly in 96.9% of users/buyers, knowledge regarding this medication may be considered as already sufficiently attained.

The women in our study declared that EC is most commonly used as an immediate resource to avoid pregnancy and not as a routine contraceptive. Comparable results are widely described in other similar studies, suggesting that the myth about the misuse of this pill by the users is no longer relevant [13, 20, 21].

The main reason provided for the use of EC is the breakage of the condom or its slippage inside the vagina. These incidents were related to the participants’ first sexual experiences and to the inability to correctly use a condom. Condom breakage seems to be a repeated theme in other studies, including a review performed by these authors [22] as well as in other international studies [6, 12, 23].

In addition, some participants mentioned that after a coital relationship, they were sometimes unsure that they had used the condom correctly, or they were not certain that they had used it at all. These perceptions were usually associated with the consumption of alcohol and/or cannabis. The use of alcohol and cannabis has been associated with risky sexual practices, possibly due to decreased inhibition and a lower sensation of risk [24, 25] which are, in turn, factors related to the use of EC [26].

Regarding women’s knowledge and misconceptions about EC, our findings coincide with previous results found by several authors [10, 11, 27]. A lack of knowledge about the mechanism of action of EC is present both in those who have used the pill and in those who have not. The lack of knowledge about the mechanisms of the drug in preventing conception or implantation may be why many women maintain false beliefs regarding the abortive effects of EC, as observed in our study and described by other authors [11–13]. However, the relationship between EC and abortion does not appear in a similar study conducted in an immigrant population [16], in which the women interviewed totally dissociate this type of pill from abortion.

The findings of Ziebland et al. [6] are consistent with our findings about the exaggeration of the secondary effects of EC, describing the existence of beliefs regarding unwanted effects if EC is used repeatedly.

Evidence exists that the uncomplicated dispensation of EC facilitates its accessibility, leading to an increase in its use. Since the sale of this drug became regulated in Spanish pharmacies, dispensation by pharmacies has increased and delivery provided by healthcare facilities has decreased, indicating the possible persistence of barriers to access through health care services [28, 29]. Our study found that some professionals continue to impart moralistic messages about the use of EC, and even convey misleading messages about how many times it is safe to use EC. In turn, these messages reinforce feelings of guilt and false beliefs held by women, thereby hindering the proper use of EC and the consequent avoidance of pregnancy.

Intentional selection of women by key informants could have resulted in a selection bias if women with more knowledge about emergency contraception were overrepresented. In that case, the discourses of more socially depressed women, with less knowledge about the different aspects of emergency contraception, which should be considered when looking at transferability to other settings. To prevent that, the theoretical sampling tried to diversify the educational levels and occupation of the women interviewed. That only 5 of the women surveyed had first-hand experience in the use of EC may be considered a limitation, but we believe that this circumstance does not adversely affect the validity of the results. The specific issues to be investigated related to the prevalent attitudes towards EC, and all of the women interviewed had either used or heard of EC. These issues include contraceptive failures; the nature of the established communication about sexuality within families, couples and peer groups; barriers to access; and the false beliefs that exist regarding contraceptive measures such as EC. All of these discourses appeared abundantly in the accounts of the women surveyed; the data are plentiful in this regard and seem to be internally consistent. Moreover, we did not identify differences in discourses between the EC users and non-users. This consistency could exist because non-users have commonly shared in the experiences of EC use by their peers. This exploratory analysis was part of a sequential mixed methods study about the emergency pill. The identification of themes and concepts has been useful in obtaining additional information about the factors related to the use of EC for inclusion in a quantitative study.

The results of this study suggest the following public health implications, which are summarized in the following points, depending on whether they apply to young people, health professionals or parents:

The sexual-affective health education received by young people should incorporate some of the findings of this work, such as the mechanism of action of the EC pill, to debunk false beliefs and misconceptions that may function as obstacles to its proper use. We must also design women’s empowerment strategies to address guilt and moralistic messages, to promote detailed knowledge about the side effects of EC, to educate about prevention of errors in condom use and to address gender stereotypes. The use of a pedagogy based on meaningful learning (based on the experiences of young people) would be desirable; in addition to increasing knowledge, such a technique may also further the development of appropriate attitudes and skills.

The design of programmes and training activities aimed at health professionals, including community pharmacists, may also be necessary to improve the level of care that is currently provided. The tendency to exaggerate the side effects of EC should be reduced, as should the communication of moral messages for the purpose of encouraging fear and guilt; these messages represent a barrier to the responsible use of this medication. We propose that public health policies should influence how these messages are transmitted.

In this respect, the contributions that are presented herein can serve as a starting point to approach the discourse of young women and to shape education and policies regarding sexual-affective health.

## Abbreviations

A: Answer
COIBA: Official College of Nursing of the Balearic Islands
EC: Emergency contraception
iDisBA: Balearic Islands Health Research Institute
MAF: MAntònia Font-Oliver
ME: Magdalena Esteva-Cantó
MI: Maria Ingla-Pol
MLB: M Lluc Bauzà-Amengual
Q: Question
SM: Sebastià March
